# Remote health monitoring for elderly through interactive television

**DOI:** 10.1186/1475-925X-11-54

**Published:** 2012-08-21

**Authors:** Susanna Spinsante, Ennio Gambi

**Affiliations:** 1 Department of Information Engineering, Marche Polytechnic University, Via Brecce Bianche 12, Ancona, Italy

## Abstract

**Background:**

Providing remote health monitoring to specific groups of patients represents an issue of great relevance for the national health systems, because of the costs related to moving health operators, the time spent to reach remote sites, and the high number of people needing health assistance. At the same time, some assistance activities, like those related to chronical diseases, may be satisfied through a remote interaction with the patient, without a direct medical examination.

**Methods:**

Moving from this considerations, our paper proposes a system architecture for the provisioning of remote health assistance to older adults, based on a blind management of a network of wireless medical devices, and an interactive TV Set Top Box for accessing health related data. The selection of TV as the interface between the user and the system is specifically targeted to older adults. Due to the private nature of the information exchanged, a certified procedure is implemented for data delivery, through the use of non conditional smart cards. All these functions may be accomplished through a proper design of the system management, and a suitable interactive application.

**Results:**

The interactive application acting as the interface between the user and the system on the TV monitor has been evaluated able to help readability and clear understanding of the contents and functions proposed. Thanks to the limited amount of data to transfer, even a Set Top Box equipped with a traditional PSTN modem may be used to support the proposed service at a basic level; more advanced features, like audio/video connection, may be activated if the Set Top Box enables a broadband connection (e.g. ADSL).

**Conclusions:**

The proposed layered architecture for a remote health monitoring system can be tailored to address a wide range of needs, according with each patient’s conditions and capabilities. The system exploits the potentialities offered by Digital Television receivers, a friendly MHP interface, and the familiar remote control, to make the service effective and easy to use also for elderly people.

## Background

Population ageing is a worldwide phenomenon, with remarkable effects on social and economic sustainability, especially in developed countries. Studies have shown that ageing has the strongest impact on long-term healthcare public investments, therefore, appropriate actions ensuring that current healthcare systems may cope with demographic and epidemiologic changes should be undertaken. At the same time, providing efficient and high-quality healthcare services to elderly people may be extremely expensive, for their reduced mobility, and for the need of moving medical equipments and operators. Communication-based technologies may allow elderly people, and, in general, people with limited mobility (such as small communities living in rural areas) or with impairments, to remotely access healthcare services, thus overcoming geographic barriers, and ensuring that services of similar quality may be provided to people living in different parts of a country. Despite the generally recognized need for the implementation of electronic healthcare (e-healthcare) systems, most of the solutions tested and provided up to now present some basic limitations, which are mainly due to the usually complex architecture they require, and to the fact that patients are typically not familiar or comfortable with the use of computers, or do not trust them enough, whereas medical operators need efficient and immediate tools. According to [1], e-healthcare and remote healthcare systems may have different target areas of applications, and even different definitions. In this paper, we propose a TV-based healthcare service (t-health) for assisted living, to enable elderly and mobility impaired people to easily collect some basic vital parameters values, and send them to a remote monitoring center, besides having the possibility of looking at their personal medical records. Multimedia input and output, in the form of graphics and speech delivered through the TV receiver, make the system appear less computer-like, and more attractive to users who are not computer-oriented.

Several motivations support the idea of using the TV medium for the delivery of healthcare services. The old analogue television represents the most widespread telecommunication service in the world, because of the low cost of its end-user devices, and thanks to the easiness of use of the receiver, through its remote control. On the other hand, dedicated reports [2,3] announce that, for example in Europe, around 35 million Digital Terrestrial Television (DTT) Set Top Boxes (STB) have been sold by the end of 2006, and a dramatic increase in sales is expected in the next few years, as further countries roll-out DTT services and increasingly prepare for the analogue switch-off. In other words, DTT, i.e. the last evolution of the traditional broadcasting, is currently the fastest growing technological platform in Europe. In the United States, the transition to digital TV has become mandatory in June 2009: as of April 2008, 91% of American broadcasters were already transmitting a digital signal. DTT STBs, when compliant with the Multimedia Home Platform (MHP) [4] specifications, are able to perform a number of interactive functions, which represent the most evident innovation with respect to traditional, analogue television. MHP STBs may connect to data networks, to exchange information with suitably configured servers. An MHP compliant STB represents a smart receiver, able to support a subset of the functionalities usually offered by a Personal Computer, by maintaining the similarity, easiness of use, and usual location of the familiar analogue receiver at the users’ premises, i.e. in front of the sofa.

Taking into account all these motivations, DTT seems the right technology to make advanced, Internet-like applications [5,6] available to a number of people that, because elderly or not learned, are not used to manage Personal Computers and devices equipped with Operating Systems [7]. A wide spectrum of experimental interactive applications based on the DTT technology, and sometimes addressing specific social groups, have been proposed, and partly tested; among them, T-learning services [8], [9], T-tourism, T-participation, and many others [10,11]. For similar motivations, part of the population may really benefit from services and applications delivered by the DVB-T technology, with the aim of allowing an easy access to healthcare facilities, and a user-friendly communication with health support centers, through a familiar medium. With personal health monitoring and data transmission, both occurring daily, patients might be able to avoid numerous trips to a physician’s surgery, and physicians could quickly act and tailor medical treatments to variations of the patient’s health situation. The proposal of delivering health assistance services over the DVB-T platform, which is investigated in this paper, aims at evaluating feasibility and effectiveness of such a solution, as a possible technological development to be accomplished in the context of assisted living.

### Related work

Distributed Diagnosis and Home Healthcare (usually denoted as *D*_2_*H*_2_) has been proposed as a new healthcare delivery framework in the 21^*st*^century. The goal of such a framework is to improve quality of care, patient wellness, and outcomes, by transforming the delivery of healthcare services from a central, hospital-based system, to one that is more distributed, patient-centered and home-based. This paradigm enforces the role played by Ambient Assisted Living (AAL) solutions targeted to elderly or impaired people. The effectiveness of the approach is confirmed, for example, in [12], where the authors discuss the benefits derived from an Internet-based telemedicine system for CardioVascular Disease (CVD) management, especially in the case of medically underserved population. Frequent surveillance improves patient care and clinical outcomes. An Internet-based telemedicine approach provides a cost effective mean to deliver preventive medicine, and a framework for chronic medical management that facilitates patient-physician communication, personalization, and education. Almost all of these services could be moved to the DVB-T platform, as suggested by the implementation herein proposed, to ensure a thorough coverage of population, even in geographical areas that may result underserved by other digital technologies. In the context of e-health, several initiatives have been undertaken in time, showing that large e-health systems may experience difficulties in managing personal data, standardizing data format, extracting content-based knowledge, and handling databases. A suitable paradigm to overcome these limitations is represented by the so called Service Oriented Architecture (SOA), which reinforces basic software architecture principles such as abstraction, encapsulation, modularization and software reuse. SOA provides welldefined interfaces for client applications, and separates interfaces from their implementations, including the policies and practices by which the services are provided and consumed [13].

Much of the work on e-healthcare systems has focused on record keeping and databases, on access and security, as well as on social implications of recording and communicating healthcare information. Recently, many efforts have been devoted also to the proper design of human computer interfaces, focusing on effective usability, which is a primary requirement for either healthcare professionals and patients. Our proposal is designed to specifically address the patients and provide user friendly input and output capabilities, with a special focus on elderly, for whom much care must be spent in designing easy-to-use and effective interfaces. In fact, the proposed system must be easy-to-use enough to be autonomously managed by the patients, without the help of a medical operator on-site. The proposed solution represents an effective way to deliver health services to the final users, through a familiar and simple medium such as television, and also to interface physicians, nurses, and medical monitoring devices outside the hospital settings.

In [14], Martinez et al. discuss information and communication needs in rural primary healthcare of underdeveloped countries. Telemedicine technologies could improve the level of these services, but the lack of infrastructures imposes strong limits to their development. Many of the rural areas have scarce or no public telecommunication infrastructure, and there are few well-trained people for management, maintenance, and repair of ICT systems. All these limitations make it necessary to resort to a low complexity solution: in this sense, the DVB-T transmissions could be provided by the national authorities, and DVB-T signal reception could be ensured at least in a few target premises, such as the main buildings where the rural communities enjoy their social activities (e.g. the town hall, the public school, or the local guard-house site). In this perspective, the solution discussed in this paper could be eventually tested as a viable opportunity even for developing countries. In general, the value of telemedicine is leveraged by leveraging ICT infrastructure. Under this point of view, our solution does not require specific advanced technology: in the basic configuration (i.e. if the return channel connection is not available), the DVB-T infrastructure can anyway provide an effective and capillary broadcast delivery of information about health diseases, risks, and their prevention, to the population. It is clear that in such a limited condition the user cannot transfer personal data to the remote health center, but it is still possible to provide a basic level of health *education* to the population. As long as a return channel connection is deployed, more advanced services may be supported on the same proposed platform.

Many efforts and proposals of integration between IT and healthcare have relied, up to now, on wireless personal communications, for example by suggesting the possibility of a wireless transfer of patient’s medical data from the ambulance to the hospital emergency room. From a technological perspective, our proposal relies on the terrestrial DVB infrastructure, to implement a home assistance service through an MHP STB. A first example of such a possibility is discussed in [15], where a telemedicine system over the DVB-T platform is presented. The proposed solution requires a so called *simplified base station* to interface the DTT STB and a 1-lead electrocardiogram through an InfraRed link. The user can manage the system by means of an interactive application loaded on the STB, to exchange data with the base station, over an RS-232 connection. The system we present differs from this solution by the important fact that the functionalities performed by the external *simplified base station* are incorporated inside the STB. This avoids the need for the user to deal with an additional device, but, most of all, it removes the RS-232 data connection, which is actually not enabled in commercial STBs (and consequently would require specific customization), due to the potential threats against the Conditional Access (CA) sub-system, that the low-level communication may allow.

A further example of an application that could be moved to the DVB-T platform may be found in [16], dealing with home automated telemanagement (HAT) for the follow-up and monitoring of patients treated with oral anticoagulant therapy. With a properly designed system architecture, health information of general interest may be broadcast, and received by all the people tuned on the right radio channel, whereas personal health information and therapy data may be received by authenticated users only, provided with a smart card, through the radio channel and/or the return channel.

Following the ideas discussed so far, this paper presents a cross-platform architecture, to provide remote health assistance based on the DTT technology, and discusses some critical features, like user privacy protection, and easy access to health and assistance information. Moreover, the proposed solution extends the traditional functions of a DTT STB, by suggesting the integration of a set of sensors for the monitoring of health parameters. By this way, the user’s STB may become a unified management device for the provisioning of remote, health-related services.

## Methods

**Figure 1:**
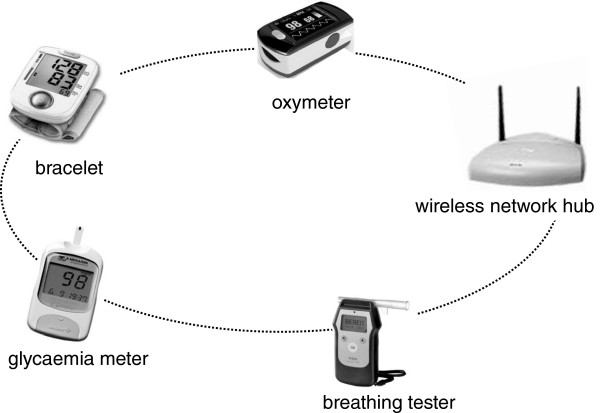
**Biological sensor network.** The network of biomedical sensors used to remotely monitor the patient’s health conditions.

The target users of the proposed system may feature a number of possible health-related problems: co-morbidity in geriatric population poses strong limitations in the patients’ lifestyle, and may greatly reduce their physical or mental independence. This motivates the need of a technological platform designed for remote health monitoring, under strict usability and accessibility criteria. An ideal remote assistance system should enable users, even older ones, the ability to easily self-monitor various health parameters, and provide important information to medical operators, thus facilitating timely healthcare decisions. The primary goal of the system herein proposed consists in collecting the patient’s data through a properly interfaced network of biological sensors, as the one depicted in Figure 1: different sensors, like oximeter, breathing tester, glycaemia meter and a wearable bracelet, equipped with a blood pressure/temperature monitor and an accelerometer for movement analysis, transfer their data through a network hub, over a wireless link, to the STB.

### System architecture and data flows

**Figure 2:**
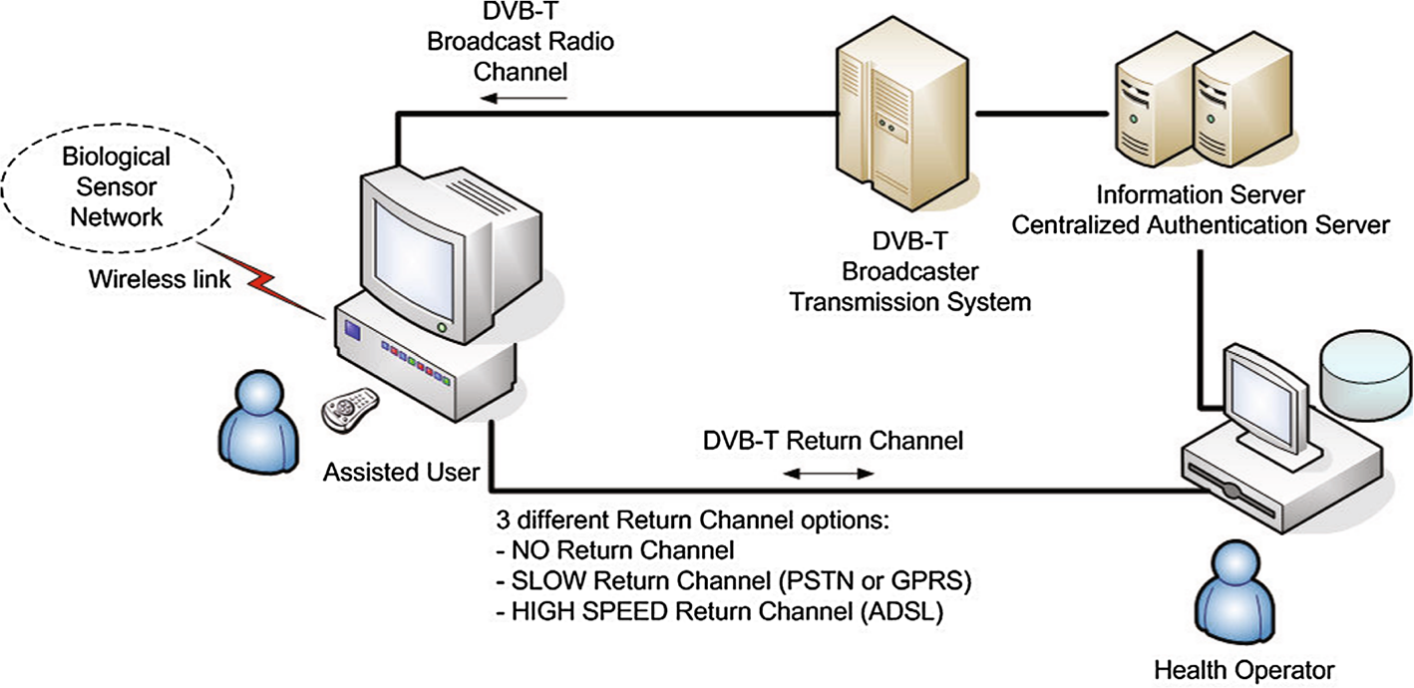
**System architecture.** The system architecture proposed for the delivery of remote healthcare services over the DVB-T platform.

The STB is able to connect to the network of sensors thanks to a USB-to-WiFi adapter that may be plugged into one of the available USB ports. In fact, commercial STBs are currently available, that support USB-to-WiFi adapters and their related drivers. Once collected by the STB, the measured data are compared with user-specific threshold values, in order to eventually activate a notification event to the health assistance centre. Such a notification is transmitted by the STB on its return channel connection. As the amount of data to transfer is limited, this service may be provided even by old STBs, equipped with a PSTN modem. It is worth to notice that the proposed system is designed according to a layered approach, i.e. it may provide different service levels, as a function of the availability (or not) and the nature of the return channel connection. In fact, according to Figure 2: 

a lower-level service is provided when the return channel is not supported. The DVB-T system conveys to the user general medical information, thanks to the broadcast nature of the communication technology chosen. In addition, user-specific information may be delivered, whose confidentiality is ensured by the adoption of smart card-based access, by exploiting the data transfer capability of DVB-T, as it will be explained in the following;

an intermediate-level service may be offered when a *slow* return channel connection is available (through a PSTN or GPRS link), that enables the possibility of exchanging data between the assisted user and the health operator;

a high-level service is finally provided when a broadband return channel connection is supported, through which a direct Audio/Video link between the patient and the health center facilities may be established, if requested.

In any case, however, the system is not designed to be used in emergency conditions, but only for long term monitoring of patients at home.

The lower-level service is actually the most critical to provide, since sending a direct feedback from the health assistance center to the patient, about the collected health parameters, is not possible. For such a reason, for each patient lacking a return channel connection, a user-specific list of threshold values for the monitored parameters (compiled by the assistance operators) is transferred to the STB over the DVB-T channel. The user’s STB, thanks to a proper MHP application, is able to interpret the received list and to compare the threshold values to the values collected by the sensors. In the case one or more parameters overcome the corresponding thresholds, the MHP application arises an alert for the user, and suggests him to contact the health assistance centre. The high level service may be supported through a classical ADSL return channel connection, for DVB-T STBs equipped with Ethernet interface, with a user bit rate capacity that may span up to 20 Mbit/s. If the cable ADSL connection is not available at the user’s premise, it may be possible to exploit a mobile broadband data connection over UMTS/HSDPA. In such a case, the user needs a mobile device acting as a router, that may interface the STB on a WiFi link, and exchange data over the UMTS/HSDPA network. In the latter scenario, the user’s STB should be chosen to support a USB-to-WiFi adapter, in order to wirelessly connect to the UMTS router device. The expected data transport capacity provided by UMTS in a static condition is enough to support the functionalities foreseen in the high level service profile. As a matter of fact, UMTS/HSDPA data connections are specifically designed to support, among the others, real time A/V connections. However, it is clear that setting up such a scenario requests the availability of a quite advanced STB, enabling USB-to-WiFi interfacing. As an alternative, a wireless return channel could be adopted, like the one provided by DVB-RCT (Return Channel Terrestrial), that offers a wireless interaction channel in the UHF/VHF bands. In the latter case, the user bit rate capacity may rise up to several Mbit/s when the wireless cell radius is reduced (e.g. from 65 Km to 3.5 Km). Both the solutions may ensure enough capacity to properly deliver A/V and data contents, in a bidirectional way, between the user and the remote health center. Similarly, also DVB-RCS (Return Channel Satellite) may support this kind of advanced service, where available (especially in rural areas) [17]. The interactive application designed for the remote health service does not depend on the specific return channel technology available, but on the available capacity to transfer different types of data.

Figure 2 shows the main blocks composing the system. At one end, the user’s premise, where the patient needing assistance lives, with the Biological Sensors Network connected over Infra Red (IR) or radio links. At the other end, the healthcare center, where the health operators, through suitable software applications, can perform a number of actions to provide differentiated levels of health assistance. The overall system is further composed by the broadcasting chain, necessary for DVB-T transmission, an Information Server (IS), and a Centralized Authentication Server (CAS) to manage the authenticated connection of each user, necessary to ensure a confidential transfer of their personal records. At the broadcaster’s premise, a proper DVB Content Manager (CM) server and a Digital Storage Media Command and Control (DSM-CC) Object Carousel (OC) server act as a repository and as a delivery engine, respectively, for the interactive and MHP-compliant applications necessary to provide additional data and services to the home user, over the DTT channel. Additionally, a Service Manager (SM) is inserted into the system architecture, to manage the remote communications (requests and responses from the STB, targeted to the various servers, and from the servers to the STB), and to link together the Web-based world, and the MHP world.

Several actors are involved in the proposed architecture: the patient, who can interact with the health operators through his DTT STB, has the possibility to authenticate by means of his personal smart card; the broadcaster, who is responsible for the delivery of audio, video and data on the radio channel, through a properly formatted MPEG-2 Transport Stream; the health operators, who can monitor each patient, send textual or multimedia messages, and provide remote assistance; finally, the healthcare centre facilities, such as the databases where each patient’s health records are stored and maintained.

**Figure 3:**
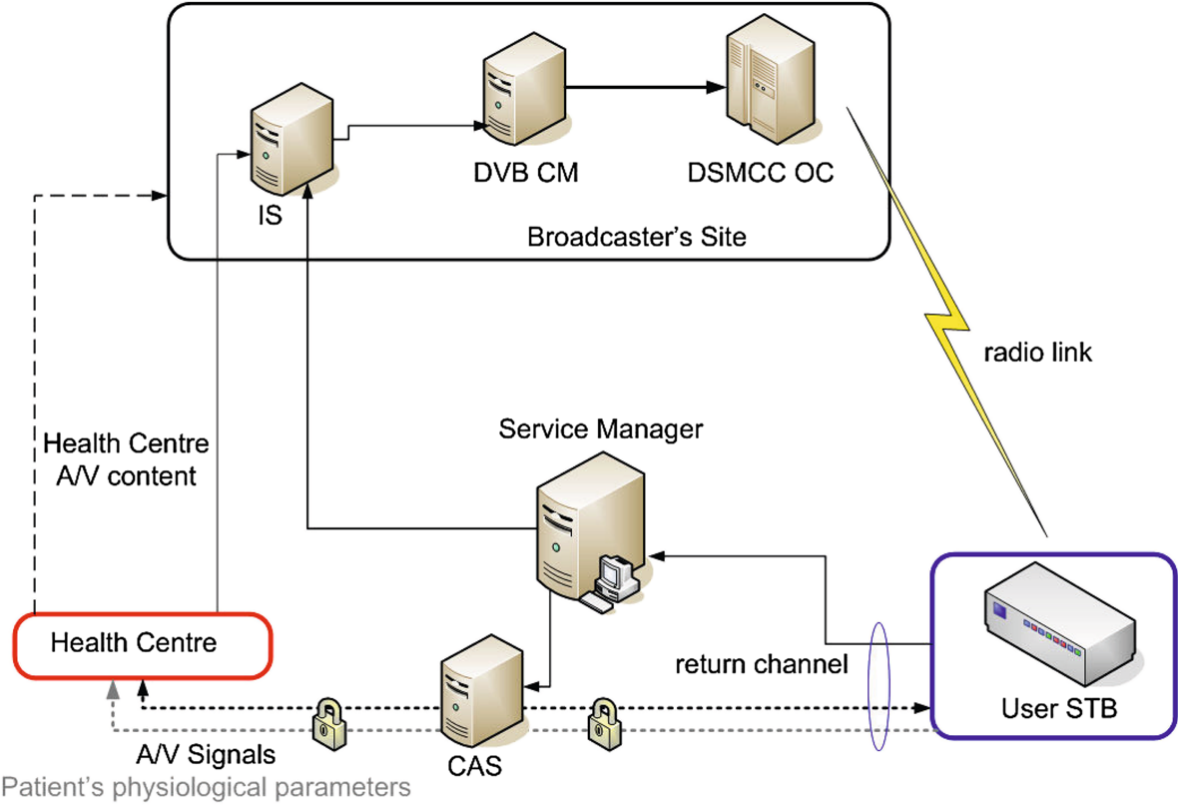
**Data flows.** The data flows exchanged among the different elements of the proposed architecture.

Figure 3 illustrates the exchange of data flows among the system elements. The user’s STB, once tuned on the proper A/V service transmitted by one of the broadcasters providing radio coverage of the area where the user lives, is able to download an interactive MHP application (i.e. an Xlet), which can offer several capabilities, like displaying information stored inside the IS, about the patient’s health history and his future medical duties, or connecting the patient to the healthcare center, through a certified procedure to establish a privacy protected link. When available, a broadband connection can be used to convey the audio signal, an optional video signal of the patient, and the information about his physiological parameters, to the healthcare centre. At the other end of the system, the IS holds the information about the patient’s health data, and acts as the content provider for the specified DVB-T service. The DVB CM transfers this information to the OC, that is responsible for the generation of the complete DVB-T Transport Stream, and for periodically broadcasting the MHP interactive application. The SM interfaced by the user’s STB on the return channel, invokes the CAS for the authentication related handshake, and enables the transfer of the user’s physiological data and A/V monitoring signals to the healthcare center, where the assistance operators may exploit the A/V connection with the user. The A/V signal of the healthcare center is privacy-protected, according with the credentials stored in the user’s smart card, either if transmitted over the DVB-T chain, and over the return channel. The assistance operators in the healthcare centre may modify or update the patient’s records stored within the IS, in order to update the ciphered information sent over the DVB-T channel along with the MHP application.

### Design of the MHP application for the health assistance service

MHP is an open standard that allows the development of interactive applications in a way which is independent from hardware platform constraints: the interface between the interactive applications and the STB operating system is given by a set of standard Application Program Interfaces (APIs). The STB middleware implements the MHP APIs in order to grant applications the access to the hardware resources in a transparent way, to handle service selection and MPEG decoding, and to execute all the operations related to Xlets and user interaction.

**Figure 4:**
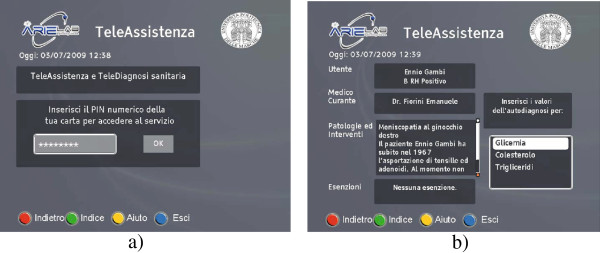
**MHP application GUI.** Screenshots of the MHP application developed for the healthcare service.

An MHP interactive application may be thought of as a set of graphic and textual pages the user can browse by pressing proper buttons on the remote control. Figure 4 shows two pages included in the graphical user interface of the Xlet designed for the proposed service. It is typical to access the Xlet environment by pressing the red button of the remote control, which makes the STB displaying the Xlet main page, like the one shown in Figure 4 a). From there, the user can navigate a number of pages related to diagnosis information, medical FAQs or health-related news, or he can start the authentication procedure by moving to the so-called authentication page. Once the authentication process is successfully completed, the user can access a number of contents about his personal health record (e.g. his health “history”, medical checks booked, or to book), as shown in Figure 4 b). Thanks to the MHP modular architecture, suited APIs can be added in time, to support emerging requirements; by this way, Conditional Access APIs and smart card management APIs have been included, to allow the authentication and privacy-protection of the personal health data exchanged. Recent evolutions in the delivery of health services by public institutions (such as in Germany, Italy, and France) brought to the adoption of Electronic Health Cards (EHC), based on the microstrip + chip smart card technology. In Italy, health services that are expected to be delivered through the new EHC will be designed according to the Italian National Services Card (NSC) framework [18]. By this way, physical compatibility between the card and the STB reader will be ensured, as DVB-T STBs are already compliant with the physical and electric standards of NSC. As a consequence, being the proposed MHP application designed to interface NSC-based smart cards, it is possible to claim the feasibility of EHC integration, if needed. Among the services that will be supported by the EHC adoption, the Electronic Health Record (EHR) will probably have a strong impact on the way citizens and public health institutions interact. In this sense, the possibility of integrating the information provided by a daily and long term monitoring service, like the one presented here, into the patient’s EHR, opens the way to a number of possible advanced services to be designed in the future.

#### The MHP application layout

**Figure 5:**
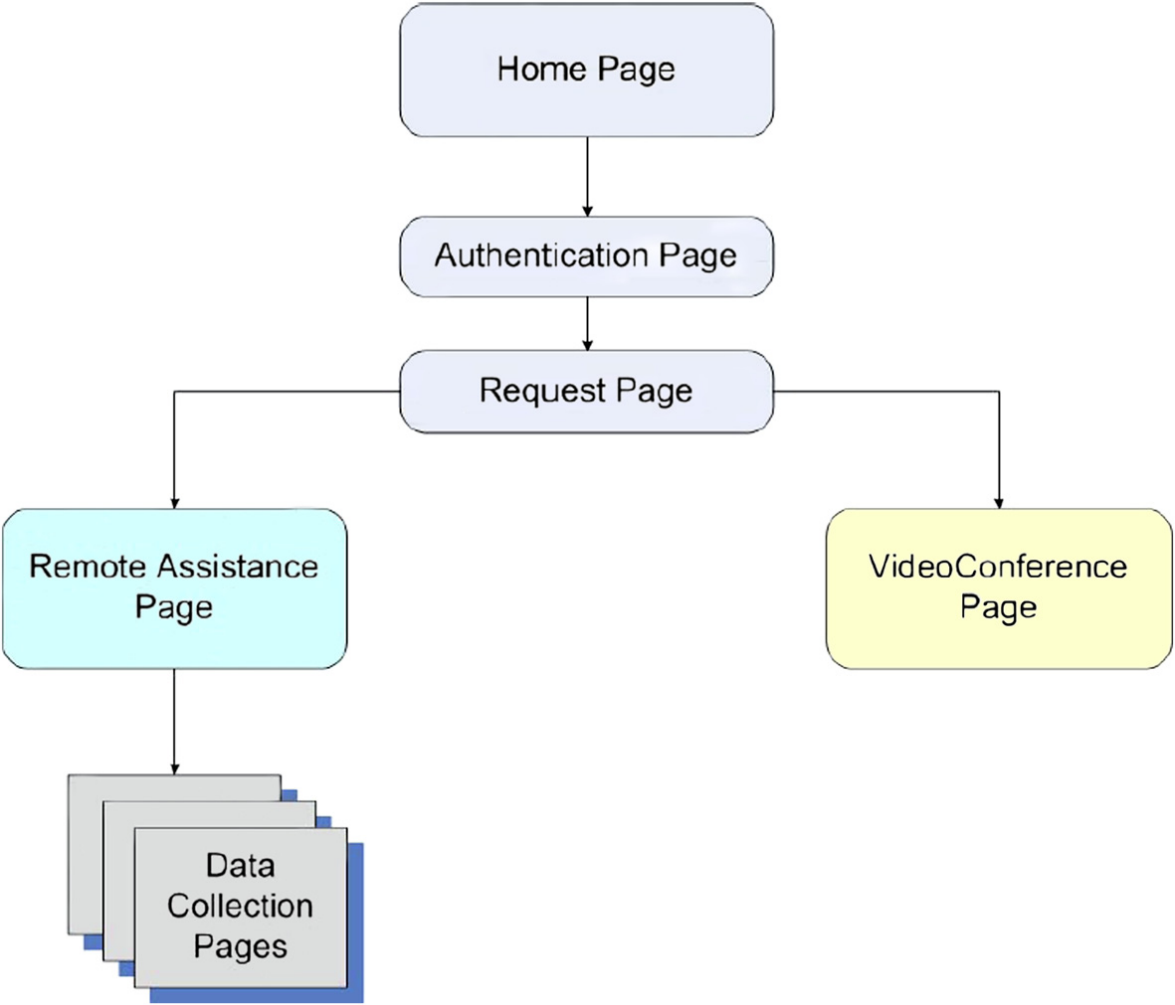
**MHP application logic.** Block diagram of the MHP application structure.

As shown in Figure 5, the MHP application has a quite simple architecture. This allows an easy identification of the main functionalities to be provided, and, accordingly, an efficient maintenance of the software application, even in view of future possible extensions. Further, the application is inspired by an SOA approach: the data can be recovered in a way independent from the technology, and treated by the execution of a specific logic. Health data collected by the sensors in the network are formatted according to a suitable XML schema, which is agreed upon by all the entities involved in the system (i.e. the Information Server, the MHP application, the health centre facilities), and they may be exchanged either over the DVB-T radio channel or the return channel connection.

First of all, the application Home Page provides the user the point of access to the service; once the user presses the red button on the STB remote control, the Authentication Page is opened. The step of user authentication, executed by inserting the user’s smart card into the proper STB slot, is fundamental in the service provisioning. It has an immediate advantage, given by the fact that the user’s personal data are collected directly from the smart card, and it is not needed to manually insert textual information by means of the remote control buttons, through a graphical keyboard. Besides that, authentication and ciphering operations are performed by the security engine implemented into the smart card, and are applied to protect sensible user data, when transferred to the healthcare centre, or to the IS located at the broadcaster’s premise. Once the authentication procedure is successfully executed, which means the user has inserted the correct Personal Identification Number (PIN), the application displays a new page, named Request Page, from which the user may choose to move to the VideoConference Page, or to the Remote Assistance Page. The former is selected when the patient wants to establish an A/V connection to the remote health centre, in order to communicate with the health operators. The latter leads the user to a number of pages dedicated to data collection: health data collected through these pages are then transferred to the remote centre over a secure connection, through the STB return channel, in a way that is transparent to the user. Different application pages deal with different health data to be collected (such as blood sugar level, blood pressure, and so on): each page is related to a specific set of data, and the graphic user interface to insert the data changes correspondingly and automatically. In the perspective of a future extension of the service functionalities, this part of the MHP application has been designed in such a way as to be able to locally save the data inserted by the user in a file, and to read them from a file. Saving data on a file is useful when data are to be transferred to the remote health centre; reading data from a file may be essential when such a file is generated by a proper appliance, that interfaces a network of biomedical devices, and collects the monitored parameters. This may represent a future extension of the service capabilities. Once the monitored data have been written and saved in a file, they are also displayed for a final check by the user, before requesting their transfer to the remote centre, by pressing a proper button on the remote control. This action activates the encryption procedure supplied by the smart card, in a way that is transparent to the user. If the user requires an A/V connection to the remote centre, the MHP application starts listening to the network interface for an UDP/RTP session; the graphic user interface manipulates the video signal to resize and place it in a proper position on the screen.

#### Authentication and privacy protection in MHP

**Figure 6:**
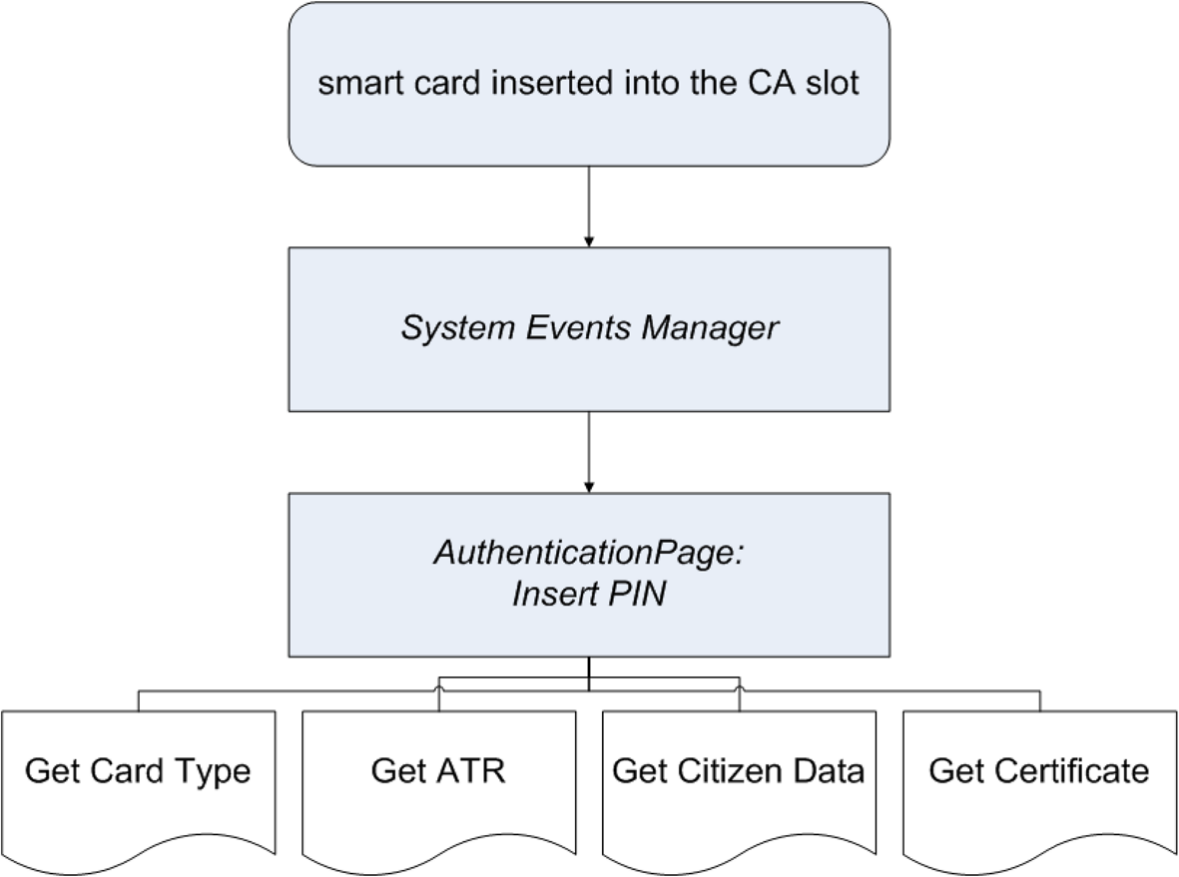
**Smart Card based Authentication in MHP.** Basic MHP procedures of the smart card-based authentication phase.

The provisioning of personal remote services through on-line platforms requires access and data transfer procedures that should be secure, easy to use, and as much general purpose as possible. Smart card based schemes can comply all these requirements, ensuring secure transactions, and privacy protection of the personal data. In the MHP environment, the Security And Trust Services API (SATSA) [19] represents the reference environment for the development of software libraries able to interface smart card devices. In the framework of the proposed service, among the smart card technologies available, the Italian NSC standard has been selected. By means of an NSC device it is possible to provide certified health, bank, postal services and others, to remote users, and univocally identify each user by means of their digital signature. An NSC is a tool for user identification in a network, and can also be used to digitally sign electronic documents. Almost all the commercial STBs provide at least one smart card reader slot for pay-per-view programs and CA systems. CA card readers are in any way compliant with the physical and electrical standards of NSC; software interfaces to smart cards, instead, have to comply with the ISO 7816-4 standard [20]. Through a suitable Java library, the application interfaces the user’s NSC. By inserting the card associated PIN, each user can access certified services, without the need of providing a username and a password for each service, but simply by plugging his card into the reader slot, as shown in Figure 6. When the user plugs the card into the CA reader slot, the STB middleware raises a specific *event* which is captured by the *EventManager* implemented by the Java MHP application, that, in its turn, causes the application to display its *AuthenticationPage*. Through this page, by means of the STB remote control, the user inputs the card PIN. Once the PIN has been typed and verified by the card, it is possible to read user’s personal data from the NSC, by accessing the *common name* field, under the *subject* section of the certificate, as reported by the Java MHP code extract:

if (state==STATE_GETCERTIFICATE) {

CNS cns = null;

X509Certificate cert = cns.getCertificate() ;

StringBuffer result = new StringBuffer();

result.append("Version: " +cert.getVersion()+"/n");

result.append("Serial Number: " +cert.getSerialNumber()+"/n");

result.append("Signature Algorithm: " +cert.getSigAlgName()+"/n");

result.append("Certification Authority:" +cert.getIssuerID()

.getName()+"/n");

result.append("Valid since: " +cert.getNotBefore()+"/n");

result.append("Valid until: " +cert.getNotAfter()+"/n");

result.append("Subject: " +cert.getSubjectDN().getName()+"/n");

⋯

} else ⋯

**Figure 7:**
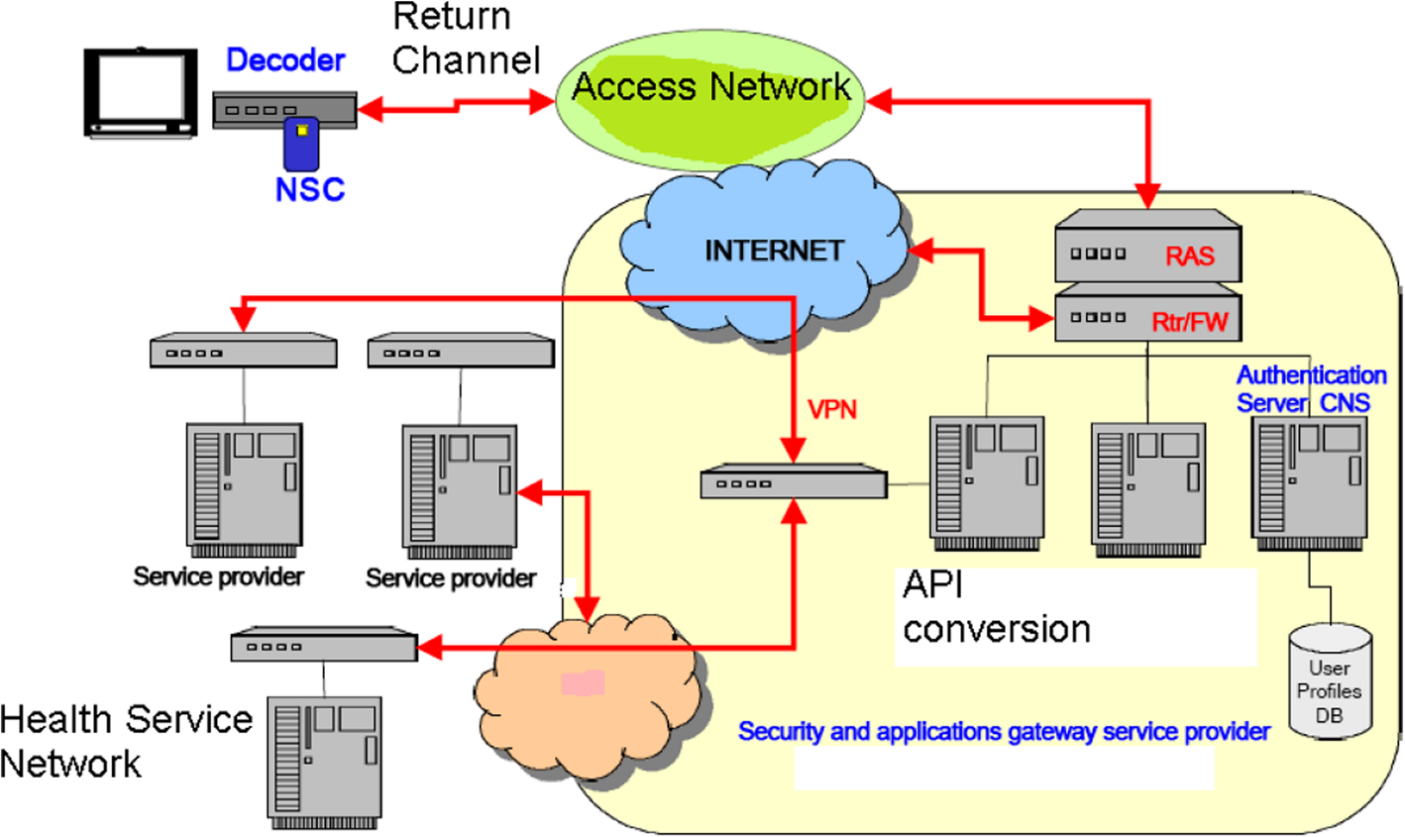
**NSC-based authentication process.** Architecture of the network infrastructure required for NSC-based security implementation.

The type of authentication described above is based on Sun X.509 v3 digital certificates, stored inside each user’s NSC device. As in X.509 v1 certificates, specific information are available, such as: serial number, certification authority identifier, card holder identity (the “Common Name” subfield contains the card holder’s tax code, that is basically used as the username in network authentication procedures), public key, expiration time, key pair generation algorithm, and others. Further, X.509 v3 certificates also include information about key usages, certificate policies, and how to query a specific certificate revocation list. When dealing with network authentication, the key usage field is set to the specific *non repudiation* value. Authentication and identification by means of MHP decoder and user NSC device is performed according to a challenge/response paradigm. The client (MHP STB) extracts from the card internal memory the user’s certificate, and sends it for authentication to the remote server; once the server verifies the client certificate, it generates a random authentication challenge and sends it back to the client. The response is computed by the client, by signing the challenge with the user’s private key stored within his NSC device. Once the response is received by the remote server, it may check the value by means of the user’s public key, that was sent inside the user’s certificate. In a successful verification, the user is authenticated. Both the user’s certificate and the health service provider certificate are issued by a recognized and trusted Certification Authority (CA). In Italy, the CA currently in charge of issuing digital certificates to promote the use of electronic authentication is the CNIPA institute, that is also responsible for the specification of the NSC framework. Thanks to the Java SATSA API, it is possible to query the NSC device for extracting the card holder information, through an MHP application. By this way, the user may avoid the manual insertion of his authentication data through the RC buttons. Figure 7 shows the authentication infrastructure used when dealing with the NSC framework. At first, the user’s connection is dealt with by the access network: the connection is directed towards the NSC authentication subsystem, and only upon successful authentication it is forwarded by the router forwarder (Rtr/FW) to the proper destination, such as the health service provider network, shown in the figure.

Another basic feature supported by MHP is the possibility to implement a secure return channel communication according with the Secure Socket Layer/Transport Layer Security (SSL/TLS) protocols, towards the services exposed by certified entities. In the context of the proposed system, this allows to set up a privacy-protected connection between the home user and the remote servers, for exchanging sensitive information. If the client (i.e. the user’s STB) needs to transfer data to the health centre, it connects to the Service Manager which redirects the connection to the CAS for authentication and authorization purposes. The authentication scheme to perform, and the cipher suite to apply, are negotiated through a proper handshake procedure. The CAS represents the core element to access any available service requiring user authentication: it checks the user credentials, by addressing a query to the User Profiles Database, and gives back the corresponding Simple Object Access Protocol (SOAP) answer. The SOAP answer is processed by the SM, converted to a suited XML file and transmitted back to the user’s STB. The Xlet parses the received XML file, and, as a consequence of a succesfull (or not) user identification, the application is enabled (or not) to use a protected connection towards the health centre.

Further details about the Java card library may be found in [21]. Automatic reading of the subject’s information stored in the card, and its use during a transaction, avoids typing by means of the STB remote control keys, that can be an action not easy to perform, particularly for elderly or impaired people.

## Results

The functions discussed above have been included in the software project developed to implement the proposed system: by this way, users that are not so familiar with typing text through the remote control, can easily provide the data requested for accessing the remote assistance service, just by inserting their smart cards. At the same time, the use of smart cards to ensure data confidentiality and user authentication could impact the system performance, by increasing the elaboration load, and consequently the time required to execute connection and security verifications through the receiver middleware. In order to evaluate the impact of smart card related operations on the timely response of the application, when requesting a remote connection through the STB return channel interface, we performed a number of experimental tests, to measure the average time needed to complete specific operations. To this aim, the MHP application has been suitably modified in order to add some check points for time acquisition, that were used to print on the screen during the application execution the time consumed by each operation under test. The experimental environment included a smart card compliant to the Italian NSC standard, equipped with a CISC processor working at 5 MHz, three commercial, MHP compliant DVB-T receivers, the main features of which are listed in Table 1, and the MHP application implementing the SATSA API, modified as stated above. At a parity of the smart card used, we expected different execution times, due to different implementations of the receiver middleware, and different hardware resources available. Evaluation of the execution times has been averaged over 10 experiments for each operation, and the values obtained are reported in Table 2.

**Table 1:** DTT receivers hardware features

**Feature**	**STB1**	**STB2**	**STB3**
Chip	STi5100	IBM PowerPC 405	STi7100
Amount of Memory	32 MB	64 MB	128 MB
Java Stack	Osmosys	Jet (open source)	Osmosys

**Table 2:** Execution times for the smart card-related operations, on different commercial STB platforms

	**STB1**	**STB2**	**STB3**
Get Data Holder	20 s	3 s	1 s
PIN Verification	3.5 s	1 s	1 s
Get Certificate	25 s	7 s	3 s

As described in Table 2, the execution of basic smart card-related functions requires, in general, a very short time, even when considering a quite limited STB platform (such as STB1). Table 2 reports the latency in the application execution due to the PIN verification procedure, which plays a basic role in the framework of the authentication procedure. This basic operation is executed in a very short time, even by STB1, and does not affect the user’s perception about the application promptness in reacting to user’s inputs. However, as the complexity of the operations involving the smart card increases, the required execution time increases as well. As an example, the amount of time needed for the Check Signature operation, which could be invoked during the secure connection to the IS, could impact negatively on the application performance. Suitable hardware resources to provide efficient smart card based services are necessary, but also properly designed MHP applications, to overcome the processing delays due to the interactions between the receiver and the card.

In the case of elderly patients, several rating criteria may be defined to assess a remote assistance system from a usability perspective. Among them: the ergonomics of the medical devices the patient should use to collect his health data (not too small dimensions, effective visual or acoustic signaling, easy switch on/off); suitably formatted and delivered instructions, to guide the user; easy and quick switch on/off of the system; clearness and easiness-of-use of the Graphic User Interface (GUI); reliability of the application used to manage the sensor devices and to collect the patient’s data (e.g. robustness against user’s misbehavior or unexpected commands, automatic restart of the application in case of unexpected crashes, robustness against lacking data connection); cognitive support and feedback messages provided by the application to the user, during the execution of different tasks; user’s satisfaction, given by the rate of successful execution of the desired task. The interactive application herein presented complies with a number of guidelines and good practices about service usability. The target audience of the proposed service is represented by elderly and/or partially impaired people: as a consequence, the design of the GUI, the fonts and colors selection, the way textual and graphic information are arranged, and the flow of operations required to interact with the system, are to be carefully evaluated and defined. As older adults progress through the natural ageing process, they may experience some degenerative effects, including reduced vision, varying degrees of hearing loss, psychomotor impairments, as well as decreasing attention, memory, and learning abilities. These phenomena affect the way older users may interact with technological equipments, and motivate the need of complying with strict design guidelines tailored to their specific needs. As an example, about the legibility of text on the screen, several recommendations are quite well known and accepted, such as: use body text not smaller than 24 points, put light text on dark background, a full screen of text should not contain more than roughly 90 words, text should be broken in small chunks that can be read almost instantly. Some of these design guidelines are summarized in [22]. The degree of usability of the application, with respect to the intended service and users, has been estimated by checking its adherence to these guidelines. Further, a preliminary experimental evaluation has been carried out, by asking a few people to try and use the application itself. Even if the number of test users, and the way they were selected (15 adults, age: 23 - 53), do not allow to generalize the preliminary outcomes obtained, it is possible to say that promising evaluations were provided, about the readability and clear understanding of the contents and functions proposed by the application. Activities are currently ongoing for obtaining a reliable evaluation of the application usability, by correctly selecting a population of test users among the target ones (elderly people possibly affected by physical impairments), and collecting their ratings through suitably structured score sheets.

Experimental tests executed in a lab setup showed that the interactive application has a stable behavior (i.e. it does not show malfunctions or crashes), and the management of the return channel interface is correctly performed. Tests on the stability and robustness of the application were performed, by simulating possible error conditions due to wrong or unintentional commands issued to the application by the user. As an example, for each graphical page presented by the application, all the buttons on the remote control were activated, in order to verify that only the expected commands could be issued, meaning that only the corresponding events raised by the STB middleware had been properly listed within the application code. It has been possible to verify that all the functionalities associated to specific buttons of the remote control were correctly executed. In a similar manner, the robustness of the application, with respect to possible unexpected events associated to the use of the return channel interface, was verified. To this aim, different error conditions were simulated, e.g. by disconnecting the network cable from the STB Ethernet interface during the operations related to the establishment of the data connection. In such a specific condition, the application raised a graphic alert to the user, in order to suggest a check of the STB connection. At the same time, as expected, the execution of the subsequent operations related to network connection was suspended. We are currently testing the sensor data acquisition procedure in a real-time context, having verified the proper data handling in an off-line configuration.

### Advanced features and future developments

Among the advanced features that could be supported by the service described in this paper (some of them are currently under development), the user may receive personalized audio messages from the remote operators over the broadcast channel. They are encoded in MPEG-1 audio format, and inserted into the MPEG-2 TS as data elementary streams. Once received by the STB, audio files are played by invoking the Java Media Framework (JMF) library supported by MHP. Personalization of audio messages may be obtained by associating to each message a tag corresponding to the destination user’s credentials, verified through his smart card. If a video communication between the patient and the health operators takes place over the return channel connection, a common WebCam (USB or IPCam) can be used, to establish an HTTP video streaming session. This service profile requires the availability of a broadband connection at the user’s premise. When video communications take place over the radio channel, care must be taken in ensuring confidentiality. It can be achieved by means of unencrypted or encrypted solutions. The former rely on the possibility of sending “phantom” A/V streams over the DVB-T channel, that can be located and MPEG-2 decoded by authorized STBs only, i.e. by STBs running a proper MHP application written ad hoc to allow phantom streams grabbing. Streams can be called “phantom” as they are included in the received A/V service, but they are not displayed by default. This strategy, adopted to protect the A/V stream, is not robust, from a strict security perspective, but provides a no-cost way to hide the desired A/V signal, when the receiving STB is not executing the proper MHP Xlet. If the user has been authenticated by means of his smart card, the MHP application locates the Elementary Streams carrying the specific phantom A/V service, and the recovered A/V signals are displayed on the user’s TV monitor. The adoption of “phantom streams” transmitted over the radio channel may allow to transfer A/V contents from the health institution to the user’s home premise, in a unidirectional fashion. Such streams shall be located within the same frequency bandwidth admitted for a DVB-T A/V broadcast channel, i.e. 6 MHz. Given the fact that the A/V quality required for the transmissions from the health center to the user’s location is not comparable to the one requested for a TV service, as the only target of “phantom transmissions” is to let the A/V signal of the health operator carry an advice or recommendation to the user (i.e. there are no diagnostic purposes in the transmission), it is possible to efficiently allocate medium-to-low quality MPEG2 encoded A/V streams within the bandwidth required by a standard definition TV channel. An alternative and advanced implementation could rely on a 6 MHz channel reserved for the delivery of health services over DVB-T.

The alternative option offers a higher level of security, as built upon the classical CA paradigm. Encryption of the A/V signal is performed to ensure its confidentiality; decryption by the user’s STB is allowed after the user’s credentials have been verified. As previously discussed, the possibility of reading the value of the monitored parameters from a file extends the range of possible future developments of the proposed system. It is possible to imagine a network of wireless sensors (i.e. biomedical monitoring devices) interfacing a proper appliance, that has the role of handling the collected data and formatting them into a suitable file. To make the data available to the MHP application, it is required to connect the appliance to the user’s STB (over a WiFi link, as an example), and to transfer the data file. The MHP application is already designed to parse and render the information included into the file, or to transfer the file to the remote health centre. Another relevant issue for the correct operation of the proposed service is related to a proper updating of the medical information provided to the users. To this aim, first of all it is necessary to define a so called Data Dictionary for each of the Data Source types foreseen by the service provider (such as health information, diagnosis). The Dictionary acts like an interface between the Xlet and the various, and different, Database schema. The process of information updating is performed in four steps: health data are inserted in the IS through a web application, by the health operators; an XML file containing such data is created and uploaded to the DVB CM; a periodic delivery of the MHP application from the DVB CM to the Object Carousel is scheduled; finally, the Object Carousel is configured to “listen to” any changes occurring in its file system. When a change happens, because new data have been inserted, the modified file system carrying a new XML file is injected into the Transport Stream. The Xlet executed by the user STB is able to parse the XML file content and display the updated information in graphical and/or textual forms.

## Conclusions

This paper discussed a Digital Television based solution for the provisioning of remote health care services to elderly or impaired people, built on a layered architecture that can be tailored to address a wide range of needs, according with each patient’s conditions and capabilities: from a low cost delivery of medical information, to an easy interface for self-health management and monitoring, to advance features, like video connection with remote operators. The proposed system exploits the potentialities offered by smart DTT receivers, a friendly MHP interface, and the familiar handling of the user STB through the remote control, to make the service effective and easy to use also for elderly people.

## Competing interests

The authors declare that they have no competing interests.

## Author’s contributions

SS provided substantial contributions to conception and design of the proposed solution, and to manuscript editing; EG has been involved in the system design, and in revising the manuscript critically. Both the authors read and approved the final manuscript.
